# Increasing Incidence of Hospital-Acquired and Healthcare-Associated Bacteremia in Northeast Thailand: A Multicenter Surveillance Study

**DOI:** 10.1371/journal.pone.0109324

**Published:** 2014-10-13

**Authors:** Maliwan Hongsuwan, Pramot Srisamang, Manas Kanoksil, Nantasit Luangasanatip, Anchalee Jatapai, Nicholas P. Day, Sharon J. Peacock, Ben S. Cooper, Direk Limmathurotsakul

**Affiliations:** 1 Mahidol-Oxford Tropical Medicine Research Unit, Faculty of Tropical Medicine, Mahidol University, Bangkok, Thailand; 2 Department of pediatrics, Sappasithiprasong Hospital, Ubon Ratchathani, Thailand; 3 Department of pediatrics, Udon Thani Hospital, Udon Thani, Thailand; 4 Center for Tropical Medicine, Nuffield Department of Clinical Medicine, University of Oxford, Oxford, United Kingdom; 5 Department of Microbiology and Immunology, Faculty of Tropical Medicine, Mahidol University, Bangkok, Thailand; 6 Department of Medicine, Cambridge University, Addenbrooke’s Hospital, Cambridge, United Kingdom; 7 Department of Tropical Hygiene, Faculty of Tropical Medicine, Mahidol University, Bangkok, Thailand; Curtin University, Australia

## Abstract

**Background:**

Little is known about the epidemiology of nosocomial bloodstream infections in public hospitals in developing countries. We evaluated trends in incidence of hospital-acquired bacteremia (HAB) and healthcare-associated bacteremia (HCAB) and associated mortality in a developing country using routinely available databases.

**Methods:**

Information from the microbiology and hospital databases of 10 provincial hospitals in northeast Thailand was linked with the national death registry for 2004–2010. Bacteremia was considered hospital-acquired if detected after the first two days of hospital admission, and healthcare-associated if detected within two days of hospital admission with a prior inpatient episode in the preceding 30 days.

**Results:**

A total of 3,424 patients out of 1,069,443 at risk developed HAB and 2,184 out of 119,286 at risk had HCAB. Of these 1,559 (45.5%) and 913 (41.8%) died within 30 days, respectively. Between 2004 and 2010, the incidence rate of HAB increased from 0.6 to 0.8 per 1,000 patient-days at risk (p<0.001), and the cumulative incidence of HCAB increased from 1.2 to 2.0 per 100 readmissions (p<0.001). The most common causes of HAB were *Acinetobacter* spp. (16.2%), *Klebsiella pneumoniae* (13.9%), and *Staphylococcus aureus* (13.9%), while those of HCAB were *Escherichia coli* (26.3%), *S. aureus* (14.0%), and *K. pneumoniae* (9.7%). There was an overall increase over time in the proportions of ESBL*-*producing *E. coli* causing HAB and HCAB.

**Conclusions:**

This study demonstrates a high and increasing incidence of HAB and HCAB in provincial hospitals in northeast Thailand, increasing proportions of ESBL-producing isolates, and very high associated mortality.

## Introduction

Nosocomial infections are the most frequent adverse event in healthcare delivery worldwide, but there is a paucity of information about their epidemiology from developing countries [1]. This is particularly true for nosocomial bacteremia which are frequently used as indicators of trends in overall nosocomial infection in developed countries because of the availability of clear definitions and clinical relevance [2], [3]. A recent comprehensive systematic review found only 13 studies of bloodstream infection from developing countries between 1995 and 2008, with only six studies in the Southeast Asia region and none from the Western Pacific region [1]. For example, the reported incidence rates of hospital-acquired bacteremia (HAB) through active surveillance were 1.0 per 1,000 patient-days in a district hospital in Kenya between 2002–2009 [4], and 1.2 per 1,000 patient-days in a university hospital in Iran in 2006 [5]. This lack of information is a consequence, at least in part, of the paucity of reliable surveillance systems for such outcomes in resource-limited settings. Moreover, published literature from developing countries is often from better resourced or university hospitals [4], [5], and may not provide a reliable basis for generalization to public hospitals in those countries.

In this study, we combined multiple sources of routine surveillance data including microbiology databases, hospital admission databases and the national death registry from a sample of provincial hospitals in northeast Thailand [6]. Our objectives were to demonstrate trends in incidence, antibiotic-resistance and mortality associated with HAB and healthcare-associated bacteremia (HCAB) over a seven year period.

## Materials and Methods

### Study population

Northeast Thailand consists of 20 provinces, covers 170,226 km^2^ and had an estimated population in 2010 of 21.4 million. Each province has a provincial hospital that provides care to people living within its catchment area and acts as a referral hospital for smaller district hospitals. The number of beds per provincial hospital ranges from 200 to 1000, and all provincial hospitals are equipped with intensive care units (ICUs). Severely ill patients presenting to district hospitals are often referred to provincial hospitals. Provincial hospitals are equipped with a microbiology laboratory that provides a bacterial culture service, while district hospitals normally do not have such facilities. All microbiology laboratories in provincial hospitals use standard methodologies for bacterial identification and susceptibility testing provided by the Bureau of Laboratory Quality and Standards, Ministry of Public Health, Thailand [7].

### Study design

We conducted a retrospective, multicenter surveillance study of all provincial hospitals in northeast Thailand. The data were collected as previously described [2]. In brief, the director of each hospital was contacted and given information on the study. For those hospitals that agreed to participate, data were collected from the microbiology and hospital databases between Jan 2004 to Dec 2010. Admission number (AN) was used as the record linkage between the two databases, and hospital number (HN) was used to identify individuals who had repeated admissions. The death registry for northeast Thailand between Jan 2004 to Jan 2011 was obtained from the Ministry of Interior, Thailand, and used to identify patients who were discharged from hospital and died within 30 days after discharge from the hospital. Ethical permission for this study was obtained from the Ethical and Scientific Review Committees of Faculty of Tropical Medicine, Mahidol University, and of the Ministry of Public Health, Thailand. Written consent was given by the director of the hospitals to use the routine hospital database for research. Consent was not sought from the patients as this was a retrospective study, and the Ethical and Scientific Review Committees approved the process.

### Data collection

The microbiology laboratory data collected were HN, AN, specimen type, specimen date, culture result, and antibiotic susceptibility profile (antibiogram). Hospital data were collected from the routine in-patient discharge report (Report 501), which is regularly completed by attending physicians and reported to the Ministry of Public Health, Thailand, as part of national morbidity and mortality reporting system. The data collected were HN, AN, national identification 13-digit number, gender, age, admission date, discharge date, and outcome. A single outcome variable is required by this reporting system, which is completed by the attending physicians and categorized as cured, improved, not improved, transfer to another hospital, refusal of treatment, or died. Date of death was also extracted from this record. Data collected from the death registry obtained from the Ministry of Interior were national identification 13-digit number and date of death. Data are not suitable for public deposition due to ethical restrictions. Raw database requests may be made to the director of each participating hospital (Table S1).

### Definitions

Bacteremia was classified as community-acquired bacteremia (CAB), HAB or HCAB. CAB was defined as the isolation of a pathogenic organism from blood taken in the first 2 days of admission and without a hospital stay in the 30 days prior to admission [2]. HAB was defined as the isolation of a pathogenic organism from blood taken after the first 2 days of admission [8], [9]. HCAB was defined as the isolation of a pathogenic organism from blood taken in the first 2 days of admission and with a hospital stay within 30 days prior to the admission [8], [9]. Patients at risk of HCAB were those with a hospital stay within 30 days prior to the admission. Patients were considered at risk of HAB after they stayed in the hospital for more than 2 days. Because of the difficulty in establishing their clinical significance, organisms frequently associated with contamination including coagulase-negative *staphylococc*i, viridans group *streptococci*, *Corynebacterium* spp., *Bacillus* spp., *Diptheroid* spp., *Micrococcus* spp., and *Propionibacterium* spp. were excluded from the analysis [10]. Organisms that produced an extended-spectrum β lactamase (ESBL) were defined using standard methodologies for bacterial identification and susceptibility testing provided by the Bureau of Laboratory Quality and Standards, Ministry of Public Health, Thailand [7]. All patients with bacteremia caused by *B. pseudomallei* were categorized as CAB because this organism is not a cause of HAB or HCAB [11]. Polymicrobial infection was defined in patients who had more than one species of pathogenic organisms isolated from the blood during the same episode. Information on patients with a first CAB episode has been published previously [2]. In this study, patients with a first episode of HAB or HCAB were evaluated in relation to epidemiology and mortality.

The 30-day mortality of HAB was determined on the basis of a record of death within 30 days of the positive blood culture taken as recorded in the routine hospital database or by a record of death in the national death registry. The 30-day mortality of HCAB was defined as death within 30 days of the admission date. The incidence rate of HAB was calculated as the number of HAB per 1,000 patient-days at risk. The cumulative incidence of HCAB was calculated as the number of HCAB per 100 readmissions. To avoid the assessment of multiple outcomes for a single patient, in the event that a patient had more than one episode of bacteremia (either HAB and/or HCAB) only the first episode was included in the study.

### Statistical analysis

All analyses were performed using STATA version 12.0 (StataCorp LP, College station, Texas). Poisson regression models were used to calculated incidence rate ratios, and logistic regression models were used to calculated odds ratios. Fisher’s exact test was used to compare categorical variables. The Mann-Whitney test was used to compare continuous variables. A non-parametric test for trend was used to assess change in proportion over time and stratified by hospital (using the npt_s command in STATA).

## Results

All 20 provincial hospitals in northeast Thailand were contacted to participate in this study. Agreement was obtained from 15 (75%) hospitals, of which 10 had microbiological laboratory and hospital databases as electronic files in a readily accessible format (Figure S1). Of the 10 hospitals included in the analysis, 3 (30%) had data available for the period 2004–2010, 1 (10%) between 2006–2010, 2 (20%) between 2007–2010, 3 (30%) between 2008–2010, and 1 (10%) between 2009–2010 (Tables 1 and 2). The median bed number was 450 beds (range 300 to 1,000 beds). A total of 1,969,474 admission records from 1,372,446 patients were evaluated, of which 21,438 (1.1%) admission records had at least one blood culture positive for pathogenic organisms during admission. A total of 3,451 (16.1%) episodes were defined as hospital-acquired bacteremia (HAB), 2,302 (10.7%) episodes were healthcare-associated bacteremia (HCAB) and 15,685 (73.2%) episodes were community-acquired bacteremia (CAB). Multiple episodes of HAB and HCAB were noted in 26 and 102 patients, respectively. Only the first episodes of HAB and HCAB in 3,424 and 2,184 patients, respectively, were included in further analysis.

**Table 1:** Incidence rates of hospital-acquired bacteremia (HAB) and associated death rate between 2004 and 2010 in northeast Thailand.

Year	Total number of hospitalswith available data	Total numberof hospital admissions	Total number of hospitaladmissions at risk of HAB*	Total number ofpatients with HAB	Deaths associatedwith HAB	30-day mortalityassociated with HAB	Incidence rate for HAB(per 1,000 patient-days)
2004	3	129,376	74,272	212	90	42.5%	0.6
2005	3	138,816	79,254	292	120	41.1%	0.8
2006	4	187,812	102,948	259	100	38.6%	0.5
2007	6	241,208	129,574	366	185	50.5%	0.6
2008	9	372,564	199,154	640	281	43.9%	0.7
2009	10	453,791	239,814	840	388	46.2%	0.8
2010	10	445,907	244,427	815	395	48.5%	0.8
Overall	10	1,969,474	1,069,443	3,424	1,559	45.5%	0.7

*Patients at risk of HAB were patients who stayed in the hospital longer than 2 days.

**Table 2:** Cumulative incidence of healthcare-associated bacteremia (HCAB) and associated death rate between 2004 and 2010 in northeast Thailand.

Year	Total number ofhospitals withavailable data	Total numberof hospitaladmissions	Total number of hospital admission at risk of HCAB*	Total number of patients with HCAB	Deaths associated with HCAB	30-day mortality associated with HCAB	Cumulative incidence for HCAB(per 100 readmissions)
2004	3	129,376	7,259	86	44	51.2%	1.2
2005	3	138,816	8,266	125	41	32.8%	1.5
2006	4	187,812	10,960	157	68	43.3%	1.4
2007	6	241,209	14,234	272	117	43.0%	1.9
2008	9	372,564	22,601	435	198	45.5%	1.9
2009	10	453,790	26,969	527	206	39.1%	2.0
2010	10	445,907	28,997	582	239	41.1%	2.0
Overall	10	1,969,474	119,286	2,184	913	41.8%	1.8

*Patients at risk of HCAB were patients who had a hospital stay within 30 days prior to the admission.

### Incidence of HAB and HCAB

The average incidence rate for HAB during the 7-year study period was 0.7 per 1,000 patient-days, with an overall increase in rate over time. The incidence rate of HAB increased from 0.6 in 2004 to 0.8 per 1,000 patient-days in 2010 (p<0.001) (Table 1).

Of 1,969,474 admission records, 119,286 (10.1%) had a hospital stay within 30 days prior to admission and were at risk of HCAB. The cumulative incidence for HCAB during the 7-year study period was 1.8 per 100 readmissions, with an overall increase in the cumulative incidence over time. The cumulative incidence of HCAB increased from 1.2 in 2004 to 2.0 per 100 readmissions in 2010 (p<0.001) (Table 2). The incidence rate of HAB and HCAB varied by hospitals (Figure S2 and S3), but the overall increasing trends were observed in most hospitals.

### Demographic risk factors for HAB and HCAB

**Figure 1:**
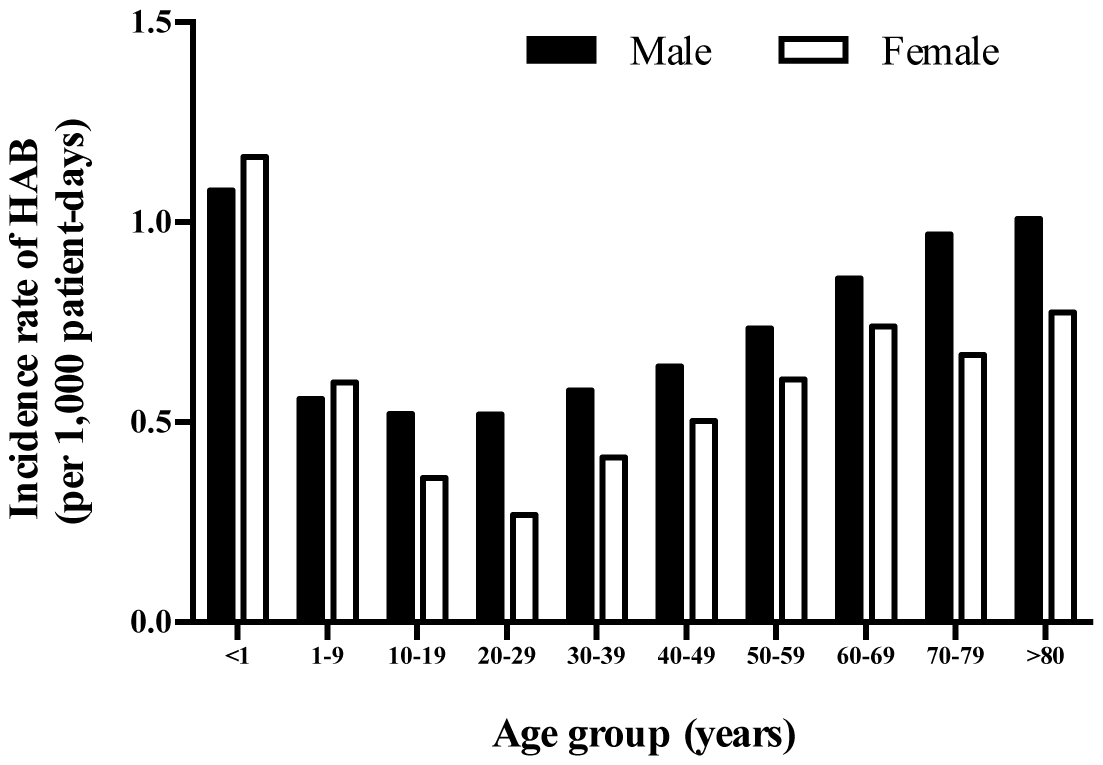
Age- and gender- specific incidence rates of hospital-acquired bacteremia (HAB) between 2004 and 2010 in northeast Thailand.

Of 3,424 patients with a primary episode of HAB, 2,000 (58.4%) were male and 1,424 (41.6%) were female. The median age was 51 years (interquartile range [IQR] 16–67 years, range 0–88 years). The median time from hospital admission to bacteremia was 8 days (IQR 4–15 days, range 3–105 days). The median length of stay for patients with HAB was longer than patients who were at risk of, but did not develop HAB (18 vs. 4 days, p<0.001). The overall incidence rate of HAB was higher in males than in females (0.8 vs. 0.6 per 1,000 patient-days, incidence rate ratio [IRR] 1.21; 95% confidence interval [CI] 1.13 to 1.30, p<0.001) (Figure 1). The incidence rates of HAB were highest in infants (1.1 per 1,000 patient-days), and in those older than 80 years of age (0.9 per 1,000 patient-days).

**Figure 2:**
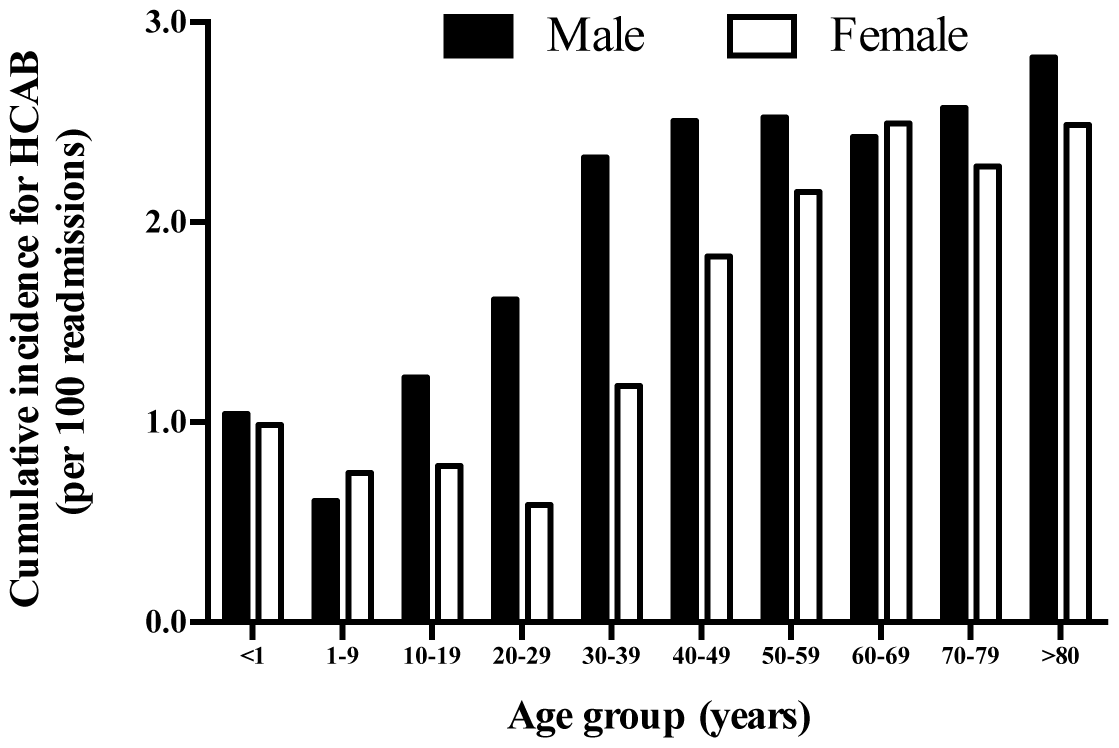
Age- and gender- specific cumulative incidence rates of healthcare-associated bacteremia (HCAB) between 2004 and 2010 in northeast Thailand.

Of 2,184 patients with a primary episode of HCAB, 1,166 (53.4%) were male and 1,018 (46.6%) were female. Median age was 57 years (IQR 41–70 years, range 0–89 years). The median time between prior and study admission was 11 days (IQR 6–19 days, range 1–30 days). The median length of stay for patients with HCAB was longer than patients who were at risk of, but did not present with HCAB (6 vs. 3 days, p<0.001). Male gender was associated with a higher risk of HCAB (odds ratio [OR] 1.29; 95%CI 1.18 to 1.40, p<0.001) (Figure 2). The incidence rates of HCAB were high in infants (1.1 per 100 readmission), and very high in those older than 30 years of age (Figure 2).

### Pathogenic organisms associated with HAB and HCAB

Of all pathogenic organisms causing HAB, 2,313 (67.6%) were Gram-negative bacteria, 885 (25.8%) were Gram-positive bacteria, 81 (2.4%) were fungi, 3 (0.1%) were *Mycobacterium* spp., and 141 (4.1%) were polymicrobial (Table 3). The most common pathogens identified were *Acinetobacter* spp. (16.2%), *Klebsiella pneumoniae* (13.9%), *Staphylococcus aureus* (13.9%), *Escherichia coli* (12.6%), and *Pseudomonas* spp. (10.5%). Amongst *S. aureus* HABs, the proportion of methicillin-resistant *S. aureus* (MRSA) was 37.0% (176/476). Corresponding proportions of extended-spectrum β lactamase (ESBL)-producing *E. coli* and *K. pneumoniae* were 38.9% (169/434) and 59.3% (283/477), respectively.

**Table 3:** Pathogenic organisms associated with hospital-acquired bacteremia (HAB) or healthcare-acquired bacteremia (HCAB).

Organisms	HAB	HCAB
Gram negative bacteria	2,313 (67.6%)	1,470 (67.3%)
*Acinetobacter* spp.	554 (16.2%)	124 (5.7%)
*Escherichia coli*		
ESBL –ve	265 (7.7%)	400 (18.3%)
ESBL +ve	169 (4.9%)	175 (8.0%)
* Klebsiella pneumoniae*		
ESBL –ve	194 (5.7%)	141 (6.5%)
ESBL +ve	283 (8.3%)	70 (3.2%)
*Klebsiella* spp.	122 (3.6%)	55 (2.5%)
*Enterobacter* spp.	155 (4.5%)	44 (2.0%)
*Pseudomonas* spp.	358 (10.5%)	205 (9.4%)
Other Gram-negative bacteria	213 (6.2%)	256 (11.7%)
Gram positive bacteria	885 (25.8%)	592 (27.1%)
*Staphylococcus aureus*		
Methicillin-susceptible	300 (8.8%)	231 (10.6%)
Methicillin-resistant	176 (5.1%)	74 (3.4%)
*Enterococcus* spp.	173 (5.1%)	74 (3.4%)
Other Gram positive bacteria	236 (6.9%)	213 (9.8%)
Fungi	81 (2.4%)	24 (1.1%)
*Cryptococcus* spp.	16 (0.5%)	20 (0.9%)
*Candida* spp.	59 (1.7%)	4 (0.2%)
*Penicillium* spp.	6 (0.2%)	–
*Histoplasma* spp.	1 (0.0%)	–
*Mycobacterium* spp.	3 (0.1%)	4 (0.2%)
Polymicrobial infection	141 (4.1%)	94 (4.3%)
Overall	3,424 (100.0%)	2,184 (100.0%)

Of all pathogenic organisms causing HCAB, 1,470 (67.3%) were Gram-negative bacteria, 592 (27.1%) were Gram-positive bacteria, 24 (1.1%) were fungi, 4 (0.2%) were *Mycobacterium* spp., and 94 (4.3%) were polymicrobial (Table 3). The most common pathogens identified were *E. coli* (26.3%), *S. aureus* (14.0%), *K. pneumoniae* (9.7%), *Pseudomonas* spp. (9.4%) and *Acinetobacter* spp. (5.7%). The proportion of ESBL-producing *E. coli,* ESBL-producing *K. pneumoniae* and MRSA were 30.4% (175/575), 33.2% (70/211), and 24.3% (74/305), respectively.

There were no differences in the patterns of common pathogens identified among different provinces or over the study period. However, there was an overall increase in the proportions of ESBL*-*producing *E. coli* over time. From 2004 to 2010, the proportion of ESBL*-*producing *E. coli* causing HAB rose from 33.3% (10/30) to 51.5% (51/99) (p = 0.005), and that causing HCAB rose from 20.8% (5/24) to 32.9% (48/146) (p<0.001). The rising trend of ESBL*-*producing *E. coli* was observed in most hospitals. We did not observe a clear overall trend in the proportions of ESBL-producing *K. pneumoniae* or MRSA.

### Mortality associated with HAB and HCAB

Death within 30-days of the positive blood culture taken was identified in 1,559 patients with HAB, giving an overall 30-day mortality of 45.5% (Table 1). Considering all patients who were admitted for more than 2 days, the 30-day mortality of those with HAB was higher than those without HAB (45.5% [1,559/3,424] vs. 5.5% [45,807/833,818], p<0.001). Death in HAB patients occurred rapidly, with 749 of 1,559 deaths (48.0%) occurring within two days of the bacteremia, 89 (5.7%) on day 3, and 74 (4.8%) on day 4. Death in HAB patients occurred in hospital in 58.4% (911/1,559) of cases, the reminder occurring after hospital discharge. There was no change in the 30-day mortality associated with HAB over time (p = 0.58).

Death within 30 days of admission with an episode of HCAB was identified in 913 patients, giving an overall 30-day mortality of 41.8% (Table 2). Considering all patients who had a hospital stay within 30 days prior to the admission, the mortality of those with HCAB was significantly higher than those without HCAB (41.8% [913/2,184] vs. 13.0% [15,168/117,102], p<0.001). Death in HCAB patients also occurred rapidly, with 410 of 913 deaths (45.7%) occurring within the first two days of admission, 54 (6.0%) on day 3, and 46 (5.1%) on day 4. Death in HCAB patients occurred in hospital in 43.2% (394/913) of cases, the reminder occurring after hospital discharge. There was no change in the 30-day mortality associated with HCAB over time (p = 0.36).

## Discussion

Our study showed that nosocomial infection is an increasing and important problem in northeast Thailand. The total number of deaths associated with HAB and HCAB in 2010 in our study (n = 634) were much higher than the total number of reported deaths due to important notifiable diseases such as dengue hemorrhagic fever (n = 139), influenza (n = 126), and leptospirosis (n = 43) during the same period countrywide [12]. There was a 32.3% increase in the incidence rate of HAB and 66.8% in the cumulative incidence of HCAB between 2004 and 2010 in northeast Thailand. These estimates reinforce the need for improved surveillance and prevention of nosocomial infection in developing countries.

An incidence rate of HAB in the participating hospitals in 2010 of 0.8 per 1,000 patient-days is higher than recent estimates in high-income countries, including 0.7 per 1,000 patient-days in Canada between 2007–2010 [13], 0.6 per 1,000 patient-days in the USA in 2005 [14], and 0.6 per 1,000 patient-days in Estonia between 2004–2005 [15]. The Thai data are consistent with a recent review showing that other parameters used to estimate the burden of nosocomial infection in developing countries, such as prevalence of healthcare associated infections and ICU-acquired infections, are substantially higher than in developed countries [1]. The recent surveillance study conducted by the International Nosocomial Infection Control Consortium (INICC) also found that rates of central line associated bloodstream infection (CLAB) were significantly higher in ICUs in developing countries (6.8 per 1,000 central line-days) versus those reported in US ICUs (2.0 per 1,000 central line-days) [16]. Our HAB incidence rate is, however, lower than HAB rates reported through active surveillance in some developing countries, including 1.0 per 1,000 patient-days in Kenya between 2002–2009 [4], and 1.2 per 1,000 patient-days in Iran in 2006 [5]. It is possible that active surveillance may improve the detection of HAB and nosocomial infection in our geographical region.

During the study period, *Acinetobacter* spp. was the most common pathogen associated with HAB, followed by *K. pneumoniae* and *S. aureus*. *Acinetobacter* spp. is increasingly recognized as an important cause of nosocomial infection [17], and our study confirms the importance this species as a leading cause of nosocomial infection in developing tropical countries [1], [4], [18]. The proportion of MRSA causing HAB in our setting (37%) was higher than that reported from developed countries [13], [15], [19], and is consistent with a previous review of developing countries [1]. An increase in the proportion of ESBL-producing *E. coli* causing HAB in our study is alarming, and is consistent with our previous report of an increase in the proportion of ESBL-producing organisms causing CAB in the same setting [6].

This study highlights an increasing incidence of HCAB in developing countries. We used the total number of patients with readmission as a denominator to estimate the cumulative incidence of HCAB rather than the total number of patients with bacteremia [8], [9], [20]. Our estimates showed that healthcare-associated infection was an increasing cause of readmission. The high proportion of MRSA, ESBL-producing *E. coli* and ESBL-producing *K. pneumoniae* amongst organisms causing HCAB was relatively similar to that causing HAB. Much lower resistance levels were seen in organisms causing CAB [6]. This is consistent with previous reports of HCAB in developed countries [8], [9], [20].

The observed increase in incidence of both HAB and HCAB could be due to a combination of an increase in the incidence of nosocomial infection associated with a rise in the number of at-risk patients (for example aging patients and those with invasive interventions), and an increase in detection of HAB and HCAB due to improved healthcare practice over time. There is evidence that the incidence rate of CLAB in developing countries can be substantially reduced using a multi-dimensional infection control approach including a bundle of interventions, education, outcome surveillance, process surveillance, feedback on CLAB rate and performance feedback [21]–[27].

The overall 30-day mortality with HAB of 45.5% in our setting is much higher than that typically reported in high-income countries [13], [15], [28], but lower than the reported in-hospital mortality of 53% from a rural district hospital in Kenya [4]. The overall 30-day mortality with HCAB of 41.8% in our setting is also much higher than typically seen in high-income countries [8], [9], [20]. In addition to patient-related factors, the higher mortality typically seen in developing countries may be related to the proportion of antimicrobial-resistant pathogens, empirical antibiotic regimens used, and sub-optimal severe sepsis management in resource-limited settings [4], [29]. It is also possible that practice in high-income countries can detect milder bacteremia cases such as cases due to intravenous device that is then rapidly removed, while the practice in low-income countries may be less likely to achieve this. The high mortality observed in our study also reflects post-dischage ascertainment of patient outcomes using the national death registry. We found that in 47.2% of fatal cases of HAB or HCAB death occurred after hospital discharge. This reflects a preference amongst people in the study area to die at home. Further studies need to explore how to reduce the mortality of patients with HAB and HCAB in resource-limited areas.

A limitation of this study is that more complete clinical data were not available. As data on central line days were not available, the incidence rate of CLAB per 1,000 central line days could not be estimated and benchmarked against other prospective studies [30]–[47]. As data on process surveillance were not available, the reasons for the increased incidence of HAB could not be systematically assessed [32], [41], [48], [49]. Another potential limitation is that blood cultures may not have been performed for all patients with a likelihood of nosocomial infection, and this might lead to an underestimation in the incidence of HAB and HCAB among participating hospitals. In addition, data on hospitalization in other hospitals not participating in the study (for example, a district hospital or a private hospital in the province) were not available, which could have resulted in an underestimation of the incidence of HAB and HCAB in our study. It is also possible that some patients with HAB and HCAB in our study may have had contaminated cultures and were incorrectly counted. However, the high mortality in patients with HAB and HCAB suggested that true infection was more likely than culture contamination. Although our data showed that, in general, patients with HAB and HCAB stayed in the hospital longer than those without, the analysis did not take account of the high mortality associated with HAB and HCAB. The length of stay would be further extended if death of patients with HAB and HCAB could be reduced. Additional costs and extra length of stay attributable to HAB and HCAB will be further evaluated using health economic models [50], [51].

Although monitoring of nosocomial infection in developing countries is hampered by incomplete routine notification, our study has shown that careful evaluation of readily available routinely collected databases can provide valuable information on the incidence and trend of HAB and HCAB. The methodology used in our study could be applied to other geographical areas where microbiological facilities are available to provide a more comprehensive global picture of the importance of nosocomial infection as a cause of death.

## Supporting Information

Figure S1 **Location of participating hospitals.** These were situated in: (1) Loei, (2) Udon Thani, (3) Nong Khai, (4) Nakhon Phanom, (5) Chaiyaphum, (6) Mahasarakarm, (7) Yasothorn, (8) Buriram, (9) Sisaket, and (10) Ubon Ratchathani.
(TIF)

Figure S2 **Trend in hospital-acquired bacteremia (HAB) in ten provincial hospitals in Thailand.**
(TIF)

Figure S3 **Trend in healthcare-associated bacteremia (HCAB) in ten provincial hospitals in Thailand.**
(TIF)

Table S1 List of participating hospitals.
(DOCX)
