# Supplementary figures and images for: Increasing Incidence of Hospital-Acquired and Healthcare-Associated Bacteremia in Northeast Thailand: A Multicenter Surveillance Study
Source: PLoS One. 2014 Oct 13;9(10):e109324. doi: 10.1371/journal.pone.0109324 (PMC4195656; doi:10.1371/journal.pone.0109324)

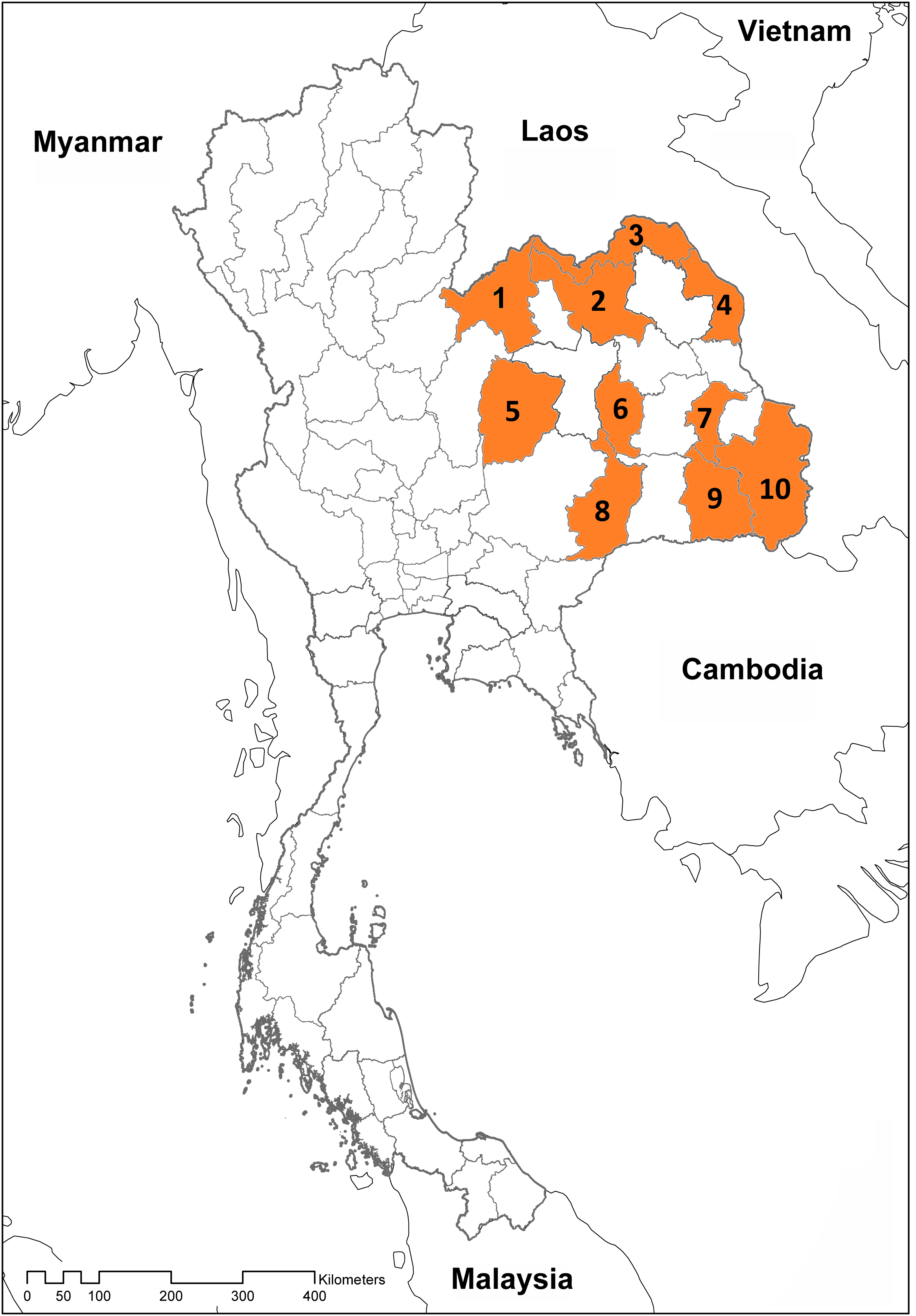

Supplement: Figure S1 — Location of participating hospitals. These were situated in: (1) Loei, (2) Udon Thani, (3) Nong Khai, (4) Nakhon Phanom, (5) Chaiyaphum, (6) Mahasarakarm, (7) Yasothorn, (8) Buriram, (9) Sisaket, and (10) Ubon Ratchathani. (TIF) [file pone.0109324.s001.tif]

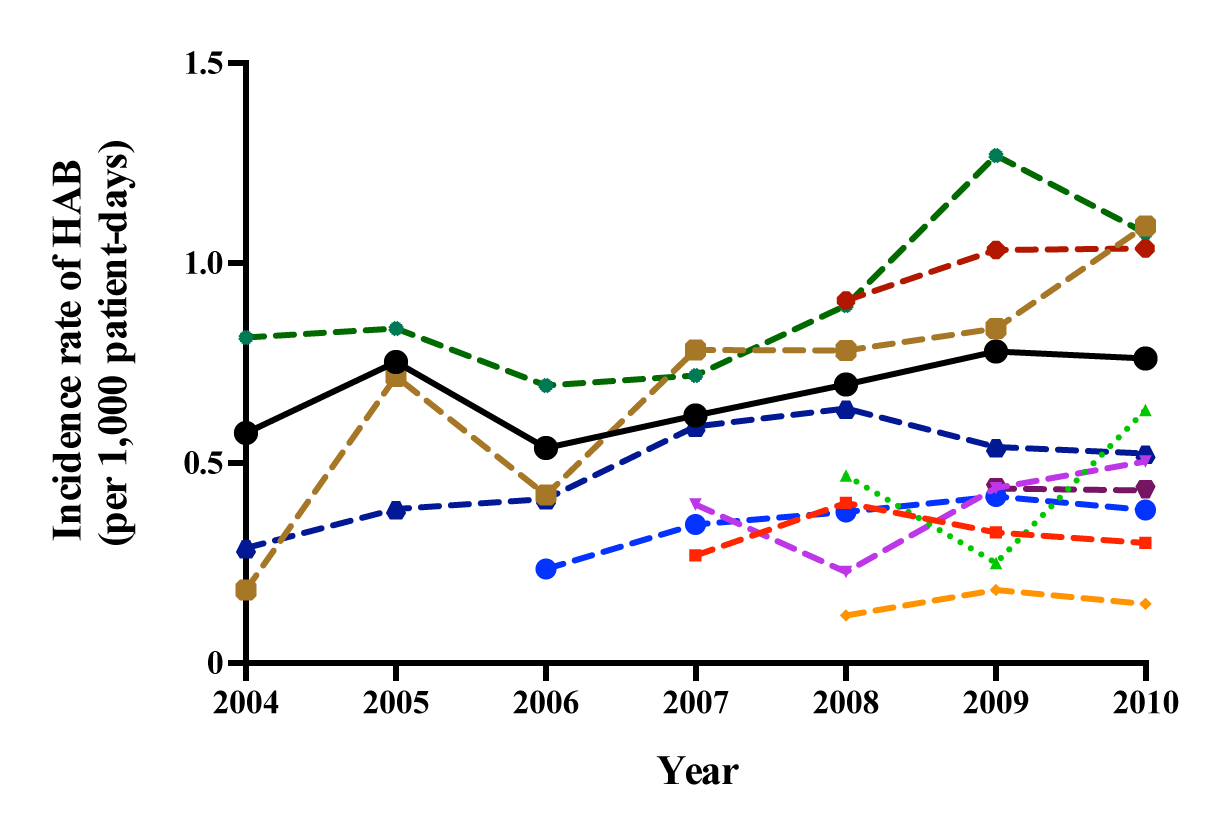

Supplement: Figure S2 — Trend in hospital-acquired bacteremia (HAB) in ten provincial hospitals in Thailand. (TIF) [file pone.0109324.s002.tif]

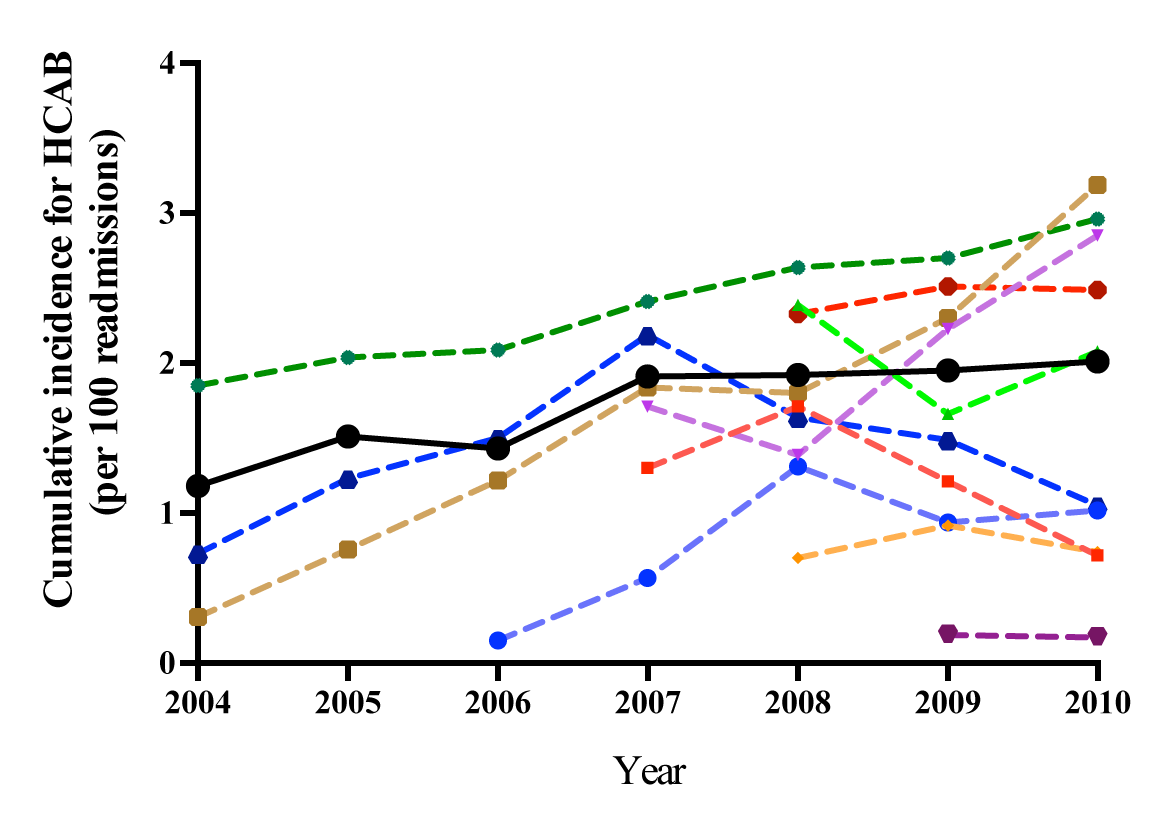

Supplement: Figure S3 — Trend in healthcare-associated bacteremia (HCAB) in ten provincial hospitals in Thailand. (TIF) [file pone.0109324.s003.tif]
