# Supplementary material for: Increasing Incidence of Hospital-Acquired and Healthcare-Associated Bacteremia in Northeast Thailand: A Multicenter Surveillance Study
Source: PLoS One. 2014 Oct 13;9(10):e109324. doi: 10.1371/journal.pone.0109324 (PMC4195656; doi:10.1371/journal.pone.0109324)
Supplement: Table S1 — List of participating hospitals. (DOCX) [file pone.0109324.s004.docx]

**Table S1.** List of participating hospitals

| Names of participating hospitals | Directors of the hospitals |
| --- | --- |
| 1. Buriram hospital | Chalit Thongprayoon |
| 1. Chaiyaphum hospital | Sompong Charoenwat |
| 1. Loei hospital | Pramoth Boonjian |
| 1. Mahasarakham hospital | Sunthorn Yontrakul |
| 1. Nakhon Phanom hospital | Somkid Suriyalert |
| 1. Nong Khai hospital | Kittisak Danwiboon |
| 1. Sisaket hospital | Udom Petpuwadee |
| 1. Ubon Ratchathani hospital | Manas Kanoksil |
| 1. Udon Thani hospital | Pichart Dolchalermyuttana |
| 1. Yasothorn hospital | Charan Thongthap |
